# Breaking the cycle: Reversal of flux in the tricarboxylic acid cycle by dimethyl fumarate

**DOI:** 10.1212/NXI.0000000000000562

**Published:** 2019-04-22

**Authors:** Arie R. Gafson, Constantinos Savva, Tom Thorne, Mark David, Maria Gomez-Romero, Matthew R. Lewis, Richard Nicholas, Amanda Heslegrave, Henrik Zetterberg, Paul M. Matthews

**Affiliations:** From the Division of Brain Sciences (T.T., R.N., P.M.M.), Department of Medicine, Imperial College, London; St Edmund Hall (C.S., P.M.M.), Oxford University, Oxford, UK; MRC-NIHR National Phenome Centre (M.D., M.G.-R., M.R.L.), Department of Surgery and Cancer, Imperial College; University College London Queen Square Institute of Neurology (A.H., H.Z.); UK Dementia Research Institute, University College London (A.H., H.Z.), London, UK; Department of Psychiatry and Neurochemistry (H.Z.), Institute of Neuroscience and Physiology, the Sahlgrenska Academy, the University of Gothenburg; Clinical Neurochemistry Laboratory (H.Z.), Sahlgrenska University Hospital, Mölndal, Sweden; and UK Dementia Research Institute at Imperial College (P.M.M.), London.

## Abstract

**Objective:**

To infer molecular effectors of therapeutic effects and adverse events for dimethyl fumarate (DMF) in patients with relapsing-remitting MS (RRMS) using untargeted plasma metabolomics.

**Methods:**

Plasma from 27 patients with RRMS was collected at baseline and 6 weeks after initiating DMF. Patients were separated into discovery (n = 15) and validation cohorts (n = 12). Ten healthy controls were also recruited. Metabolomic profiling using ultra-high-performance liquid chromatography mass spectrometry (UPLC-MS) was performed on the discovery cohort and healthy controls at Metabolon Inc (Durham, NC). UPLC-MS was performed on the validation cohort at the National Phenome Centre (London, UK). Plasma neurofilament concentration (pNfL) was assayed using the Simoa platform (Quanterix, Lexington, MA). Time course and cross-sectional analyses were performed to identify pharmacodynamic changes in the metabolome secondary to DMF and relate these to adverse events.

**Results:**

In the discovery cohort, tricarboxylic acid (TCA) cycle intermediates fumarate and succinate, and TCA cycle metabolites succinyl-carnitine and methyl succinyl-carnitine increased 6 weeks following treatment (q < 0.05). Methyl succinyl-carnitine increased in the validation cohort (q < 0.05). These changes were not observed in the control population. Increased succinyl-carnitine and methyl succinyl-carnitine were associated with adverse events from DMF (flushing and abdominal symptoms). pNfL concentration was higher in patients with RRMS than in controls and reduced over 15 months of treatment.

**Conclusion:**

TCA cycle intermediates and metabolites are increased in patients with RRMS treated with DMF. The results suggest reversal of flux through the succinate dehydrogenase complex. The contribution of succinyl-carnitine ester agonism at hydroxycarboxylic acid receptor 2 to both therapeutic effects and adverse events requires investigation.

Dimethyl fumarate (DMF) (BG-12; Tecfidera) is a fumaric acid ester licensed as a disease-modifying treatment for relapsing-remitting MS (RRMS).

However, DMF is best considered as a prodrug. After oral administration, it is rapidly hydrolyzed by esterases in the small intestine to monomethyl fumarate (MMF).^1^ MMF is highly bioavailable, has a half-life of 12 hours, and reaches peak concentrations of approximately 20 μM. MMF itself is hydrolyzed inside cells to fumaric acid, which initiates secondary metabolism of the drug through the tricarboxylic acid cycle (TCA).^2,3^

Common adverse events including flushing and gastrointestinal symptoms (abdominal pain and diarrhea) limit the tolerability of DMF by some people with RRMS.^4^ Metabolites of DMF including MMF are believed to be responsible for the primary therapeutic effects through activation of the transcription factor nuclear factor (erythroid-derived 2)-like 2 (Nrf2),^5,6^ inhibition of nuclear factor κB,^7^ and/or agonism of the hydroxycarboxylic acid receptor 2 (HCA2, GPR109A).^8^ Additional metabolites also could mediate these and other effects. Although flushing, diarrhea, and nausea all could arise from HCA2 agonism, the specific mechanism responsible for adverse events associated with DMF has not been defined.

We have sought to better characterize the specific molecular effectors of therapeutic effects and adverse responses after DMF administration through untargeted metabolomics. This approach can help characterize drug metabolism^9–11^ or identify biomarkers relevant to drug effects^12^ through multivariate correlation of metabolic features and clinical measures.^13^ These data enable generation of new hypotheses concerning therapeutic benefits of drugs or associated adverse events.^11^

Here, we have used separate small groups of people with RRMS who were newly initiating treatment with DMF to characterize short-term (6 week) metabolomic pharmacodynamic effects to infer possible major molecular effectors of therapeutic responses and relate these to adverse events. We used a first group for discovery and then tested these outcomes in a separate validation group. We also related the results to a biomarker for axonal injury in MS, plasma neurofilament light (NfL).

## Methods

### Standard protocol approvals, registrations, and patient consents

Our research study was reviewed and approved by the NREC Committee of London Camden and Islington (NREC 14/LO/1896). All patients provided written informed consent.

### Study design

This study included a previously described cohort of patients with RRMS^14,15^ separated into an initial discovery cohort and a validation cohort to test for the generalizability of results. Patients diagnosed with RRMS by the McDonald criteria^16^ were recruited from the Imperial College Healthcare NHS Trust and consented for participation in the study. Patients recruited were aged between 18 and 65 years and treatment-free (disease-modifying treatments [DMTs] and steroids) for at least 3 months. Previous work had demonstrated that metabolome effects of this drug are large,^17^ so the sizes of the discovery and validation cohorts could be small. Ten age- and sex-matched healthy volunteers were recruited as controls by local advertising and did not receive any treatment.

The discovery cohort included 15 patients with RRMS (median Expanded Disability Status Scale [EDSS] score 1.5, range 1–6.5; additional clinical information provided in table e-1 links.lww.com/NXI/A106). The validation cohort included 12 patients with RRMS (median EDSS score 3.0, range 1–7). The patients and healthy volunteer controls both attended the study center for 2 visits. For the patient cohort, this was at baseline, before onset of treatment, and 6 weeks after commencement of treatment with DMF. For the healthy volunteer cohort, there were also 2 study visits at 6-week intervals, but no drug was taken. The EDSS score was assessed on all the patients at each of their visits by a single, trained physician (A.R.G.). Detailed information on adverse events was taken from direct questioning and clinical histories obtained by a single, trained physician (A.R.G.).

### Sample collection

Nonfasting venous blood samples were collected at study visits in ethylenmdiamine tetraacetic acid tubes and centrifuged at 1,400*g* for 10 minutes within 3 hours of sample collection. Plasma was separated immediately into aliquots of 1 mL and stored at −80°C.

### Ultra-high-performance liquid chromatography mass spectrometry

#### Discovery cohort

Samples were sent to Metabolon Inc (Durham, NC) for untargeted metabolomic analysis. Samples were precipitated with methanol, followed by centrifugation before addition of several control samples to aid chromatographic alignment. This included pooled matrix samples, technical replicates (derived from a pool of well-characterized human plasma), process blanks, and within-sample spiking of endogenous compounds. Experimental samples were randomized across the platform and run with the control samples described above.

All methods used a Waters ACQUITY ultra-performance liquid chromatography (UPLC) and a Thermo Scientific Q-Exactive high-resolution/accurate mass spectrometer interfaced with a heated electrospray ionization-II) source and Orbitrap mass analyzer operated at 35,000 mass resolution. Samples were analyzed using 3 UPLC-tandem mass spectrometry (MS/MS) assays: acidic positive ion conditions (optimized both for hydrophilic and hydrophobic compounds), basic negative ion conditions, and a negative ionization following elution from a hydrophilic interaction liquid chromatography (HILIC) column. The scan range covered 70–1,000 m/z.

Raw data were extracted and peaks identified using the Metabolon library. Biochemical identifications are based on 3 criteria: retention index within a narrow retention index window of the proposed identification, accurate mass match to the library ±10 ppm, and the MS/MS forward and reverse scores between the experimental data and authentic standards. Peaks were quantified using area under the curve.

#### Validation cohort

To determine whether findings seen in the discovery cohort could be replicated, UPLC-MS assays were performed in a validation cohort analyzed by the National Phenome Centre (Imperial College, UK). Samples were prepared as previously described.^18^ A single HILIC UPLC-MS analysis was performed on an Acquity UPLC instrument coupled to a Xevo G2-S oaTOF mass spectrometer (Waters Corp, Manchester, UK) via a Z-spray electrospray ionization (ESI) source operating in the positive ion mode. Details of the UPLC-MS system configuration and HILIC analytical method used for profiling have been reported previously.^19^ Feature extraction and data processing were performed using Progenesis QI 2.1 software (Waters Corp,) as previously described.^19^ Metabolites of interest from the discovery cohort analysis were located either by retention time and accurate mass match to an authentic reference standard or by accurate mass and interpretation of the MS/MS fragmentation pattern (specifically for methyl succinyl-carnitine, as no reference standard was commercially available).

### Targeted quantitative analysis of MMF

Targeted analysis for the absolute quantification of MMF concentrations was performed for all samples. Briefly, samples were prepared by dilution with 3 volumes of acetonitrile + 0.1% formic acid containing 100 mg/mL heavy labeled MMF (mono-methyl-^13^C,*d*_3_ fumarate, Sigma-Aldrich). The samples were mixed and centrifuged as described above before solid-phase extraction using OSTRO sample preparation plates (Waters Corp, Milford, MA) operated by vacuum manifold for 2 minutes. The product sample was dried overnight under a continuous flow of nitrogen gas and reconstituted using an amount of ultra-pure water equal to the original volume of plasma used (150 µL).

Sample analysis was performed using an Acquity UPLC instrument coupled to a Xevo TQ-S tandem quadrupole mass spectrometer (Waters Corp, Manchester, UK) via a Z-spray ESI source operating in the negative ion mode. MMF was identified using the National Phenome Centre reference library as being well retained by the reversed-phase chromatographic method described previously^19^ and that method was therefore validated with the following parameters: limit of detection = 0.5 ng/mL; limit of quantification = 5 ng/mL; linear range = 0.5–100 ng/mL; dynamic range = 0.5–2000 ng/mL; sensitivity = 0.99 ± 0.023. Within-run precision was measured by 7 repeated analyses of samples at the low, medium, and high range of the method (%relative standard deviation = 3.3, 1.6, and 2.4, respectively. Matrix effects and absolute recovery covered a chosen low, medium, and high range within the dynamic range (40, 400, and 800 ng/mL). Matrix effects indicated negligible ion suppression with values above 95% with no ion enhancement. Absolute recovery was within acceptable range of 77%–118% in accordance with stated GLP and good maufacturing practice bioanalytical method validation guidelines. Peak integration and calculation of the final MMF concentration were performed using TargetLynx software (Waters Corp).

### Plasma NfL

Plasma NfL concentration was measured using a commercially available digital ELISA on a Single Molecule Array instrument as described by the kit manufacturer (Quanterix, Lexington, MA). The measurements were performed in 1 round of experiments using 1 batch of reagents. Intra-assay coefficients of variation were <10%.

### Statistical analyses

Descriptive statistics were used to summarize MS patient and healthy control demographics (table 1). The significance of changes in specific metabolites pre- and post-treatment were estimated relative to comparisons with data from the healthy volunteer controls acquired over the same period using 1-way analysis of variance. All changes were corrected for multiple comparisons using the false discovery rate. Statistical significance was set at a q value of <0.05. Correlation between discriminant variables of interest and concentrations of MMF was performed using the Pearson correlation coefficient. Comparison of absolute NfL concentrations at different time points was analyzed using the paired Student's *t* test. Comparisons of NfL between patients with MS and healthy controls was performed using an unpaired, 2-tailed Student *t* test.

**Table 1:**
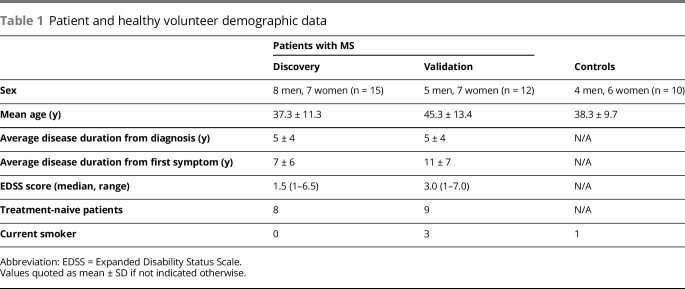
Patient and healthy volunteer demographic data

To discover the most discriminatory variables from the data set pre- and post-treatment in the first of our 2 patient cohorts, we used the Random Forest method.^20^ We used 1,000 trees and 5-fold cross-validation to build the model. A variable importance measure was computed based on the mean decrease accuracy metric. All statistics were performed in R.

### Data availability statement

Anonymized data will be shared by request from any qualified investigator.

## Results

Patient demographics and clinical information are provided in table 1, with their detailed medical history in table e-1 links.lww.com/NXI/A106. Ten patients with RRMS were initiating treatment with DMF after a minimum period of 3 months since treatment with any previous DMT. Seventeen patients were treatment naive to previous DMT. None of the healthy volunteer controls reported comorbid disease or current medical treatments.

### Concentrations of TCA cycle intermediates and their metabolites are increased after administration of DMF

Concentrations of both fumarate and succinate were increased in the samples obtained from patients in the discovery cohort 6 weeks after the start of treatment (q < 0.05). Concentrations of succinyl-carnitine and methyl succinyl-carnitine, which are synthesized from succinyl-Coenzyme A (CoA), also were increased (q < 0.05) (table 2, figure, A–D). Significant changes in concentrations of these metabolites were not seen in a contrast of baseline and 6-week plasma samples from the untreated healthy control population.

**Table 2:**
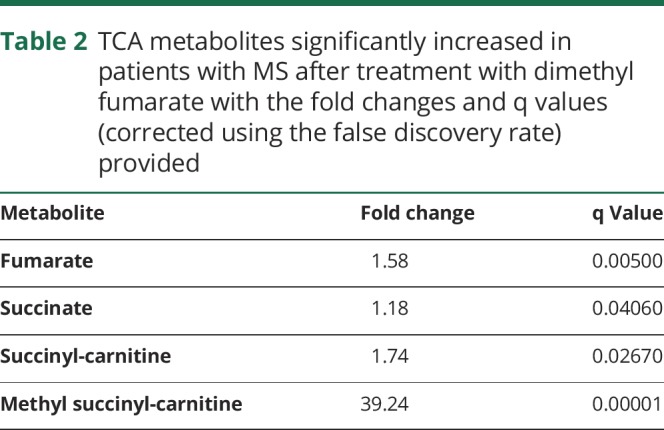
TCA metabolites significantly increased in patients with MS after treatment with dimethyl fumarate with the fold changes and q values (corrected using the false discovery rate) provided

**Figure:**
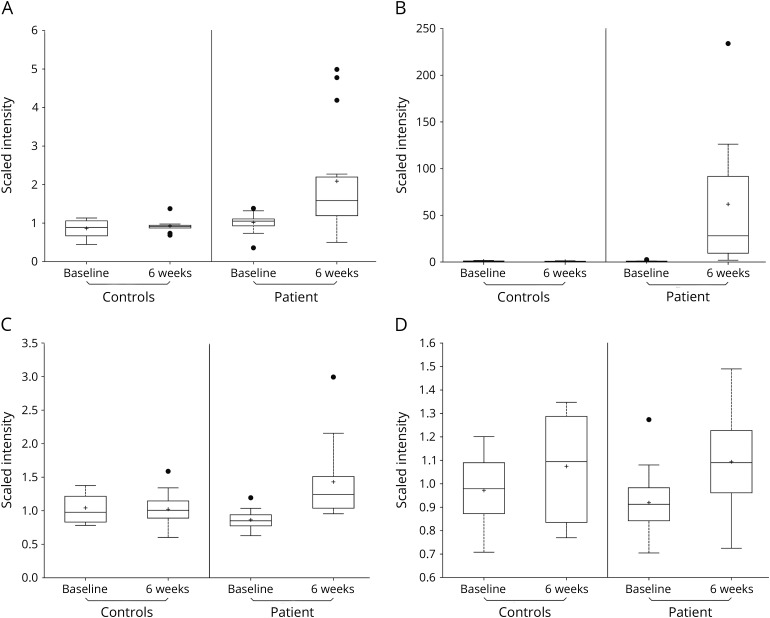
(A–D). Boxplots of metabolites best discriminating effects of treatment (A, succinyl-carnitine; B, methyl succinyl-carnitine; C, fumarate; D, succinate) at baseline before treatment with dimethyl fumarate and 6 weeks after starting treatment in patients from the discovery cohort (treated) relative to the untreated healthy volunteer controls

We then performed a Random Forest analysis to discover those metabolites whose changes in concentrations best discriminate patients after treatment relative to their DMF-naive states. Concentration changes in methyl succinyl-carnitine, fumarate, and succinyl-carnitine best discriminated patient samples 6 weeks after the start of treatment from those at baseline. The mean decrease in accuracy (MDA) with their individual contributions was derived by excluding each metabolite in turn from the model with separate calculations for the decrease in accuracy of the classification. For methyl succinyl-carnitine, fumarate, and succinyl-carnitine, the MDA was 10.2, 8.6, and 6.0, respectively (table 3).

**Table 3:**
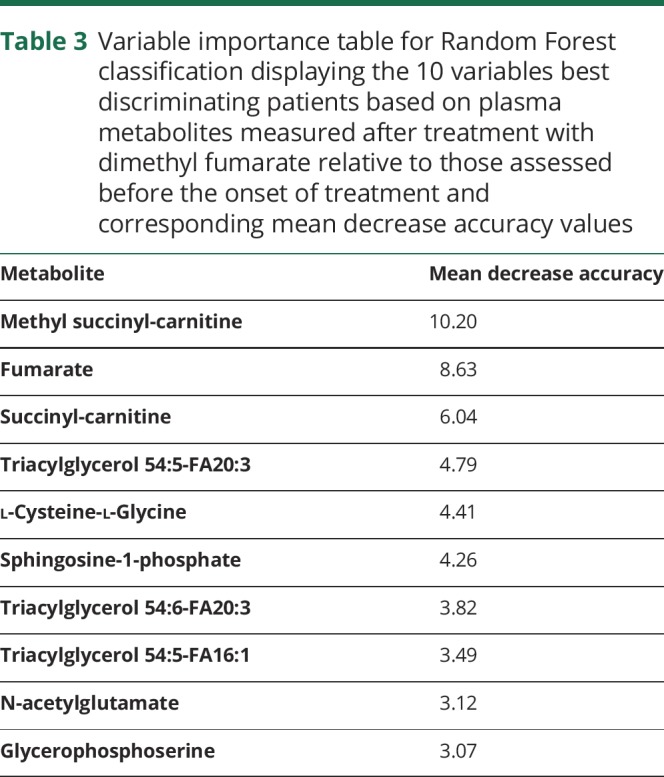
Variable importance table for Random Forest classification displaying the 10 variables best discriminating patients based on plasma metabolites measured after treatment with dimethyl fumarate relative to those assessed before the onset of treatment and corresponding mean decrease accuracy values

We sought to confirm the association of DMF treatment with increased concentrations of TCA cycle intermediates and their metabolites in a separate validation cohort. The most significantly increased metabolite in the validation cohort also was methyl succinyl-carnitine (retention time_mass/charge ratio 4.71_276.1448), which changed by 145-fold (*p* < 0.005). Succinyl-carnitine, fumarate, and succinate were not observed in the single UPLC-MS method used to analyze the validation data set.

We measured concentrations of MMF, the major primary metabolite of DMF, in plasma samples. We found plasma concentrations in the patients clustered into 2 distinct groups: patients had either very low (<20 ng/mL) or high (range 95–592 ng/mL) concentrations of MMF. The Pearson correlation showed a strong relationship between plasma concentrations of methyl succinyl-carnitine and MMF (r = 0.66). Levels of MMF did not correlate with patient-reported timing of the last dose (r = −0.19).

### Association between common adverse events and concentrations of succinyl-carnitine and methyl succinyl-carnitine

Gastrointestinal symptoms (abdominal pain, diarrhea, and nausea) and flushing were common adverse events (21/27 in the total cohort). We contrasted the annotated metabolites in the baseline and 6-week samples in the discovery cohort from those who experienced these adverse events with those who did not. In patients experiencing flushing after DMF administration (8/15) (table e-1, links.lww.com/NXI/A106), there was a significant increase in methyl succinyl-carnitine after DMF (fold change 42.60 ± 44.33, *p* < 0.05) and a trend toward a significant change in succinyl-carnitine at 6 weeks (fold change 1.92 ± 1.07, *p* = 0.06). These changes in the 6-week relative to baseline samples were not found in those not experiencing flushing (*p* = 0.08 and *p* = 0.17, respectively).

In those experiencing abdominal symptoms (6/15) (table e-1, links.lww.com/NXI/A106), a significant change was found for both methyl succinyl-carnitine (fold change 180.49 ± 206.83, *p* < 0.05) and succinyl-carnitine (fold change 3.81 ± 1.52, *p* < 0.05) plasma concentrations at 6 weeks. In those not experiencing these symptoms, there was a smaller change in methyl succinyl-carnitine concentration (fold change 27.53 ± 33.78, *p* < 0.05) and no change in succinyl-carnitine (*p* = 0.12).

### Association between DMF treatment and plasma NfL concentration

We investigated the effects of DMF treatment on plasma NfL concentrations at short- (6 weeks) and medium-term (15 months) treatment periods relative to untreated pretreatment levels. Mean plasma NfL concentrations were similar in the pretreatment (13.2 ± 18.56 pg/mL) and 6-week posttreatment (14.1 ± 22.25 pg/mL) samples. These mean values were more than twice the mean concentrations in the healthy volunteers sampled according to the same schedule (5.94 ± 1.92 pg/mL, baseline, and 5.83 ± 2.51 pg/mL, 6 weeks), although the differences were not statistically significant between groups. There was an approximately 40% mean reduction in the NfL concentration (7.83 ± 3.94 pg/mL) in the patients 15 months after initiation of treatment. No meaningful changes in plasma NfL were observed in the untreated healthy volunteer control group at 15 months (6.88 ± 3.67 pg/mL) relative to the earlier time points.

## Discussion

DMF is metabolized to MMF and is believed to exert its therapeutic effects through antioxidant and anti-inflammatory pathways. These in turn may modulate both innate and adaptive immune processes independently of Nrf2.^21^ However, the precise molecular mechanisms are not well understood. Here, we used global metabolomics profiling of blood plasma to better define the metabolism of DMF. Using mass spectrometry, the greatest changes observed were seen in TCA cycle intermediates fumarate and succinate and in the secondary TCA cycle metabolites succinyl-carnitine and methyl succinyl-carnitine. The potential anti-inflammatory properties of these metabolites (and their association with adverse effects) suggest that reversal of TCA cycle flux through succinate dehydrogenase may be crucial to the pharmacodynamic properties of DMF.

One previous study investigating the acute effects (24 and 72 hours) of DMF on a human oligodendrocyte cell line also reported increases in succinate and fumarate with treatment.^22^ Furthermore, others have confirmed that administration of DMF in vitro causes a rise in the concentration of succinate.^23,24^ To our knowledge, ours is the first study of its metabolic effects in patients with MS. Our results confirmed an increase in the concentrations of these TCA cycle intermediates in plasma after initiation of treatment with DMF. We also found significant increases in succinate esters (succinyl-carnitine and methyl succinyl-carnitine) with treatment.

Carnitine esters, found in high concentrations, previously have been shown to have a variety of potentially beneficial effects and may be important mediators of therapeutic responses. l-carnitine and acetyl-l-carnitine have both been demonstrated to activate antioxidant pathways mediated by Nrf2.^25–27^ Furthermore, in a rat model of interstitial nephropathy, reduction in 2 acyl-carnitines resulted in impaired Nrf2 pathways and activation of NF-κB, suggesting that acyl-carnitines may exert their effects independently of Nrf2.^28^ Carnitine esters can readily cross the blood-brain barrier (BBB)^29^ and are known to have neuroprotective^30^ and anti-inflammatory properties.^31^ Carnitine esters also increase concentrations of beta-hydroxybutyrate,^32–34^ an agonist of the HCA2 receptor (GPR109A), through activation of the urea cycle. GPR109A has been suggested as the potential mediator of both the therapeutic^35,36^ and adverse effects of DMF through its effects in peripheral immune cells.^37–39^ In this study, we found an association between flushing and increases in the concentration of succinyl-carnitine providing indirect evidence that carnitine esters may be responsible for this common adverse event associated with DMF.

The observation of an increase in succinate without increases in intermediates involved early in the forward direction of the cycle (malate, citrate, and α-ketoglutarate) suggests that fumarate generated from metabolism of DMF is reduced to succinate by reversal of flux through succinate dehydrogenase. Reversal of succinate dehydrogenase arises as a consequence of high concentrations of intracellular succinate, which can occur during ischemia.^40^ Furthermore, patients with mutations in succinate-CoA ligase, which normally catalyzes conversion of succinyl-CoA to succinate, characteristically show increased concentrations of both succinyl-CoA and succinyl-carnitine, for example.^41–44^ It remains to be determined whether the secondary succinylation of carnitines^45,46^ may be mediating the therapeutic or adverse effects of DMF; succinate itself is a potent anti-inflammatory molecule.^47^

We sought to determine whether DMF could alter plasma NfL concentration, given emerging evidence that it may be a useful marker of therapeutic response to other DMTs.^48,49^ To our knowledge, there are no published data addressing the impact of DMF on plasma NfL concentrations. Here, we have demonstrated a clear trend toward reductions in DMF over a 15-month period that was not found in healthy volunteers of a similar age. Although changes in plasma NfL were not statistically significant, there was a large variation in concentrations found in the MS cohort, and many did not have substantially increased concentrations at baseline.

A limitation of this study was the small sample size and consequent uncertainty regarding the potential to generalize from our results, given the heterogeneity in the disease and therapeutic responses. However, the primary metabolic effects of the drug, for which the study was powered, were large as expected,^17^ and we were able to replicate our most significant finding in a second validation cohort, even using a different mass spectrometry platform. A practical limitation of this and other similar biomarker research was that sampling was limited to plasma rather than also from the CNSCNS. In animal models of MS, DMF has been shown to upregulate Nrf2^5^ in the CNS specifically and also to protect neural progenitor cells from oxidative stress.^50^ However, given that DMF is rapidly hydrolyzed to MMF, it is controversial whether these effects occur in vivo. In support of the relevance of plasma measures, evidence suggests that peripheral immune cells are most likely to be the effectors of the therapeutic actions of DMF.^51^ To confront the primary limitation of the sample size, future work could study a larger treatment population with more active disease.

In conclusion, we have shown that concentrations of some TCA cycle intermediates and their metabolites are significantly increased in the plasma of patients with MS treated with DMF. The discovery of elevated succinyl-carnitine esters, which are secondary metabolites of DMF, highlights the possibility that these species may be responsible both for the therapeutic effects and adverse events associated with the administration of DMF. However, this hypothesis requires formal testing. This could be tested in vitro by application of succinyl-carnitine and methyl succinyl-carnitine to cell subsets such as dendritic cells or lymphocytes and measuring expression of genes or proteins known to be modulated by Nrf2 and NF-κB, for example. A further outstanding question is whether carnitine esters exert their effects in the periphery, the CNS, or both, given that there is evidence they can freely cross the BBB exerting antioxidant and neuroprotective effects.^33^ Further understanding of their pharmacokinetic properties and species-specific tissue distributions will assist in exploring their potential role in the therapeutic effects of DMF.

## Glossary

BBB: blood-brain barrier
DFM: dimethyl fumarate
DMT: disease-modifying treatment
EDSS: Expanded Disability Status Scale
ESI: electrospray ionization
HCA2: hydroxycarboxylic acid receptor 2
MDA: mean decrease in accuracy
MMF: monomethyl fumarate
MS/MS: tandem mass spectrometry
NfL: neurofilament light
Nrf2: nuclear factor (erythroid-derived 2)-like 2
RRMS: relapsing-remitting MS
TCA: tricarboxylic acid
UPLC-MS: ultra-high-performance liquid chromatography mass spectrometry
